# Biomarkers of PEGylated Liposomal Doxorubicin-Induced Hypersensitivity Reaction in Breast Cancer Patients Based on Metabolomics

**DOI:** 10.3389/fphar.2022.827446

**Published:** 2022-04-21

**Authors:** Wei Zhuang, Xiuping Lai, Qingxiu Mai, Suiwen Ye, Junyi Chen, Yanqiong Liu, Jingshu Wang, Siming Li, Yanqing Huang, Tao Qin, Hai Hu, Junyan Wu, Herui Yao

**Affiliations:** ^1^ Phase I Clinical Trial Centre, Sun Yat-sen Memorial Hospital, Sun Yat-sen University, Guangzhou, China; ^2^ Department of Medical Oncology, Sun Yat-sen Memorial Hospital, Sun Yat-sen University, Guangzhou, China

**Keywords:** breast cancer, PEGylated liposomal doxorubicin, hypersensitivity reaction, l-histidine, metabolomics, toxicity

## Abstract

This study aimed to analyze and discuss the biomarkers of PEGylated liposomal doxorubicin (PLD) injection-induced hypersensitivity reactions (HSRs) in advanced breast cancer patients. Fourteen patients from Sun Yat-sen Memorial Hospital were included in the study between April 15th, 2020 and April 14th, 2021. Patient plasma was collected 30 min before PLD injection. HSRs were found to occur in a total of 9 patients (64.3%). No association was found between HSRs and various patient characteristics such as age, body surface area, anthracycline treatment history, IgE, and complement 3 and 4 (*p* > 0.05). Non-targeted metabolomics analysis of patient plasma was performed, and several metabolites showed significant association with HSRs. In particular, l-histidine (fold change = 91.5, *p* = 0.01) showed significantly higher levels in the immediate HSR group, while myristicin (fold change = 0.218, *p* = 0.003), urocanic acid (fold change = 0.193, *p* = 0.007), and d-aldose (fold change = 0.343, *p* = 0.003) showed significantly lower levels in the same group. *In vivo* experiments showed that exogenous histidine aggravated HSRs and increased IgE plasma levels in rats following the injection of PLD. Histidine can be decarboxylated to histamine by histidine decarboxylase. Histidine decarboxylase inhibitor 4-bromo-3-hydroxybenzoic acid improved symptoms and IgE levels *in vivo*. These findings suggested that l-histidine can be a potential biomarker for PLD-induced HSR. Moreover, an antihistamine drug, histidine decarboxylase inhibitor, or dietary histidine management could be used as potential preventive measures. Furthermore, metabolomics research could serve as a powerful method to explore biomarkers or uncover mechanisms of drug side effects.

## 1 Introduction

According to a 2020 report by the World Health Organization’s International Agency for Research on Cancer, breast cancer had the highest number of new cases in the world (IRAC, 2020). An important ambition of cancer care in the 21st century is to recover the precancer quality of life and emotional and social functions, which is only possible through the mitigation of the side effects of anticancer treatments (Henry et al., 2012; Franzoi et al., 2021). Although anthracyclines such as doxorubicin, pharmorubicin, and pirarubicin have many side effects such as cardiotoxicity and myelosuppression, anthracyclines remain a cornerstone drug in breast cancer treatment. PEGylated liposomal doxorubicin (PLD) is an anthracycline nanomedicine approved for advanced breast cancer and other solid tumor therapy (Keller et al., 2004) and has shown a 57% disease control rate (Oostendorp et al., 2011).

Unfortunately, PLD could induce many side effects, including cardiotoxicity, myelosuppression, hair loss, hand–foot syndrome, oral mucositis, and immediate hypersensitivity (Alberts and Garcia, 1997). Various reports suggest that 9–25% of patients experience either an infusion reaction or immediate hypersensitivity reaction (HSR) (Alberts and Garcia, 1997; Szebeni et al., 2018) with symptoms such as flushing, shortness of breath, facial swelling, headache, chills, back pain, tightness in the chest or throat, or hypotension (Szebeni et al., 2018). Severe immediate HSR could lead to anaphylactic shock, presyncope, or, in some cases, even be life-threatening (Castells et al., 2008).

To our knowledge, no sensitivity biomarker has yet been identified to predict PLD-induced HSR. Furthermore, the mechanism of PLD-induced HSR is also unknown. Therefore, this study aimed to analyze the biomarkers and discuss the mechanism of PLD-induced HSR in advanced breast cancer by obtaining a comprehensive plasma metabolic fingerprint from PLD-induced HSR patients.

## 2 Materials and Methods

### 2.1 Patients and Study Design

A total of 14 advanced breast cancer patients from Sun Yat-sen Memorial Hospital were enrolled in this study between April 15th, 2020 and April 14th, 2021. The inclusion criteria were as follows: ≥18 years old; breast cancer confirmed by histological or molecular diagnosis; advanced breast cancer diagnosis according to the *American Joint Committee on Cancer Eighth Edition Cancer Staging Manual*; treatment with PLD alone; no pregnancy during the study period; and voluntary use of effective contraceptive measures during treatment. The exclusion criteria were as follows: subjects who discontinued treatment due to previous severe adverse reactions to PLD or doxorubicin; or poor compliance.

Before PLD injection, unnecessary combination treatment was stopped seven days before injection. Patients had the same breakfast at around 7 a.m. Sample collection time and PLD injection time were set at 9:30 a.m. and 10:00 a.m., respectively. Samples were collected again 30 min after injection.

Hypersensitivity was evaluated according to the Common Terminology Criteria for Adverse Events (CTCAE) 5.0.

This study was approved by the Ethical Committee of Sun Yat-sen University Memorial Hospital (SYSEC-KY-KS-2021–015). Written informed consent was obtained from all participants.

### 2.2 Sample Collection

Patients’ plasma was collected within 30 min before PLD injection and again 30 min after injection. The blood samples (3 ml each) were drawn into EDTA polypropylene tubes. And the plasma was separated by centrifugation at 4°C 1,000 g for 10 min. All samples were frozen at −80°C until analysis.

### 2.3 Sample Pretreatment

To extract total metabolites, sample pretreatment was conducted as follows: 400 µL of precooled methanol was added to 100 µL plasma samples and vortexed; then centrifuged and all supernatants were transferred and concentrated to dry in vacuum; samples were then dissolved with 150 µL 2-chlorobenzalanine (4 ppm) and 80% methanol solution; then 20 µL from each sample was taken as the quality control (QC) samples. The samples were used for liquid chromatography–mass spectrometry (LC-MS) detection.

### 2.4 Metabolomics Detection

A Thermo Ultimate 3000 system equipped with an ACQUITY UPLC^®^ HSS T3 (150 × 2.1 mm, 1.8 µm; Waters) column maintained at 40°C was used for chromatographic separation. Gradient elution of analytes was carried out with 5 mM ammonium formate in water (A) and acetonitrile (B), or 0.1% formic acid in water (C) and 0.1% formic acid in acetonitrile (D) at a flow rate of 0.25 ml/min. An increasing linear gradient of solvent B (v/v) was used as follows: 0–1 min, 2% B/D; 1–9 min, 2%–50% B/D; 9–12 min, 50%–98% B/D; 12–13.5 min, 98% B/D; 13.5–14 min, 98%–2% B/D; 14–20 min, 2% D-positive model (14–17 min, 2% B-negative model). All the samples were given a random number. The samples were injected in LC-MS/MS according to the random number.

A Thermo Q Exactive mass spectrometer was used to perform the ESI-MS assay. Positive and negative modes using spray voltage was set to 3.5 kV and −2.5 kV with 325°C capillaries and 30 eV normalized collision energy. The analyzer scanned over a mass range of *m/z* 81–1,000 for a full scan at a mass resolution of 70,000.

### 2.5 Metabolomics Data Analysis and Quality Control

#### 2.5.1 Data Preprocessing

Raw data were transformed into an mzXML format by ProteoWizard software (v3.0.8789) (Smith et al., 2006). Peak identification, filtration, and alignment were accomplished by the XCMS package of R (v.3.3.2). The mass-to-charge ratio (*m/z*), retention time, and relative ratio of the peak area were acquired. Overall, 3,787 and 12,671 precursor molecules were acquired from the positive and negative ion models, respectively.

#### 2.5.2 Quality Control and Quality Assurance (Want et al., 2010; Dunn et al., 2011)

QC samples were mixed with 20 µL from each sample. The QC samples were used to monitor deviations of the analytical results from these pool mixtures and compare them to errors caused by the analytical instrument itself. To guarantee quality, the peaks with relative standard deviation (RSD) ≤30% will be retained for the next analysis.

### 2.6 Bioinformatics Analysis

#### 2.6.1 Hierarchical Clustering

Agglomerate hierarchical clustering was used in this study. The relative quantitative levels of metabolites were determined using the Pheatmap package of R (v.3.3.2). All samples and related data were calculated using a distance matrix and clustered using the average linkage clustering method.

#### 2.6.2 Multivariate Analysis (Thévenot et al., 2015)

Autoscaling, mean centering, and scaling to unit variance (UV) were used to accomplish the multivariate analysis for scaling processing. SIMCA-P (v13.0) and R language ropls packages were used to perform principal component analysis (PCA), partial least squares-discriminant analysis (PLS-DA), and orthogonal partial least squares-discriminant analysis (OPLS-DA). The OPLS-DA results are shown in Supplementary Figure S1.

#### 2.6.3 Identification of Differential Abundant Metabolites

Differential metabolites were identified using parameters with variable importance for the projection (VIP) ≥1.00 and a *p* value <0.05. We first assure the precise molecular weight (MW) of metabolites (MW error <30 ppm). Metlin (http://metlin.scripps.edu), MoNA (https://mona.fiehnlab.ucdavis.edu), and a standard database built by BioNovoGene Co., Ltd. (Suzhou, China) were subsequently applied according to the MS/MS fragmentation pattern to check the annotation and acquire corresponding information. Then the metabolites database was built by BioNovoGene Co., Ltd., and normalization into relative content on the same level was carried out for further analysis. Agglomerate hierarchical clustering analysis was conducted, and differential metabolites were shown with the heat map. The statistical analysis of differential metabolites was showed by a Z-score map, which was calculated based on the mean and standard deviation of the control group, and expressed as Z=(*x*-μ)/s, where *x* indicates a specific score, m denotes the average, and s represents the standard deviation (Chen et al., 2015). Metabolic pathway analysis based on Kyoto Encyclopedia of Genes and Genomes (KEGG) and Metabolomics Pathway Analysis (MetPA) and referred to the hypergeometric test (Kanehisa and Goto, 2000).

### 2.7 Quantification of Plasma IgE, C3, and C4 Levels

Human IgE, C3, and C4 levels were detected by nephelometry at the Department of Laboratory Medicine, Sun Yat-sen Memorial Hospital. Additionally, rat IgE levels were detected using an ELISA kit according to the standard operating procedure.

### 2.8 *In Vivo* Experiment Design

In this study, Sprague Dawley rats weighing about 150 g were used to confirm the l-histidine and histidine decarboxylase inhibitor 4-bromo-3-hydroxybenzoic acid (BHBA) effects of PLD. There were 4 group settings (*n* = 6): control group (normal saline i.p. 1 ml treated 5 days, after 120 min, PLD i.v. 0.1 mg: 0.1 ml); histidine group (l-histidine i.p. 40 mg treated 5 days: 1 ml, after 120 min, PLD i.v. 0.1 mg: 0.1 ml); BHBA group (normal saline i.p. 1 ml treated 5 days, after 100 min, BHBA i.p 0.15 mg: 0.2 ml, after 20 min, PLD i.v. 0.1 mg: 0.1 ml); and histidine + BHBA group (l-histidine i.p. 40 mg treated 5 days: 1 ml, after 100 min, BHBA i.p. 0.15 mg: 0.2 ml, after 20 min, PLD i.v. 0.1 mg: 0.1 ml). Approximately 0.5 ml blood was collected before PLD was injected and 2 min after injection, respectively. Following euthanasia, the larynx, trachea, and lungs of the rats were collected for further analysis.

### 2.9 Staining

All tissues were fixed in 10% neutral buffered formalin and blocked with paraffin under routine processing. Pulmonary edema was identified by H&E staining. Mast cell quantification was carried out by using toluidine blue O staining.

### 2.10 Statistical Analysis

All statistical analyses were performed using SPSS version 21.0 (IBM^®^). Categorical variables were analyzed by using either the χ^2^ test or Fisher’s exact test. Continuous variables were analyzed with the Mann–Whitney *U*-test to compare two subgroups. *p* value in untargeted metabolomics results had been corrected using the Benjamini–Hochberg (BH) procedure. As for *in vivo* relative assays, differences between groups were estimated by unpaired *t*-tests or ANOVA. Statistical significance was assumed for *p* values less than 0.05.

## 3 Results

### 3.1 Patients’ Characteristics and PEGylated Liposomal Doxorubicin-Induced Immediate Hypersensitivity

Of the 14 breast cancer patients involved in this study, 9 patients (64.3%) experienced immediate hypersensitivity within 2 mins postinjection. No significant association was found between various patient characteristics, including age, height, weight, body surface area (BSA), estrogen receptor (ER), progesterone receptor (PR), human epidermal growth factor receptor-2 (HER-2), anthracycline treatment history, and anthracycline accumulated dose (Table 1). Plasma IgE, complement 3 (C3), and complement 4 (C4) levels were further detected, and 5 allergy patients demonstrated high IgE and C4 levels (IgE, C3, and C4 levels for each patient are shown in Supplementary Table S1). However, no association was found between hypersensitivity and IgE, C3, or C4 (Table 1).

**TABLE 1:** Association of patients’ characteristics and PLD-induced HSR.

	Mean (range)/positive rate/N(yes/no)	*p* value^a^	OR (95% CI)
Age (years)	50 (37, 68)	0.431	1.056 (0.922–1.210)
Height (cm)	155.5 (146.0, 165.0)	0.493	1.072 (0.879–1.307)
Weight (kg)	54.75 (46.2, 61.9)	0.320	0.884 (0.694–1.127)
BSA^b^ (m^2^)	1.60 (1.49, 1.77)	0.747	0.146 (0.000–1.73×10^4^)
ER	64.3%	0.076	12.000 (0.773–186.362)
PR	64.3%	0.076	12.000 (0.773–186.362)
HER-2	64.3%	0.803	1.333 (0.139–12.818)
Anthracycline treatment history (N, yes/no)	10/4	0.486	2.333 (0.216–25.245)
Anthracycline accumulated dose	328.45 (103.9, 496.8)	0.802	0.999 (0.993–1.005)
IgE	78.2 (4, 413)	0.338	1.009 (0.991–1.028)
C3	1,183.9 (714, 1,510)	0.998	1.000 (0.994–1.006)
C4	318.1 (154, 532)	0.893	1.001 (0.990–1.011)
Immediate hypersensitivity (N, yes/no)	9/5	—	—

a p value: Fisher’s exact test; estrogen receptor (ER); progesterone receptor (PR); human epidermal growth factor receptor-2 (HER-2).

b BSA, body surface area.

PLD, PEGylated liposomal doxorubicin; HSR, hypersensitivity reaction.

### 3.2 Metabolites and PEGylated Liposomal Doxorubicin-Induced Immediate Hypersensitivity

**FIGURE 1:**
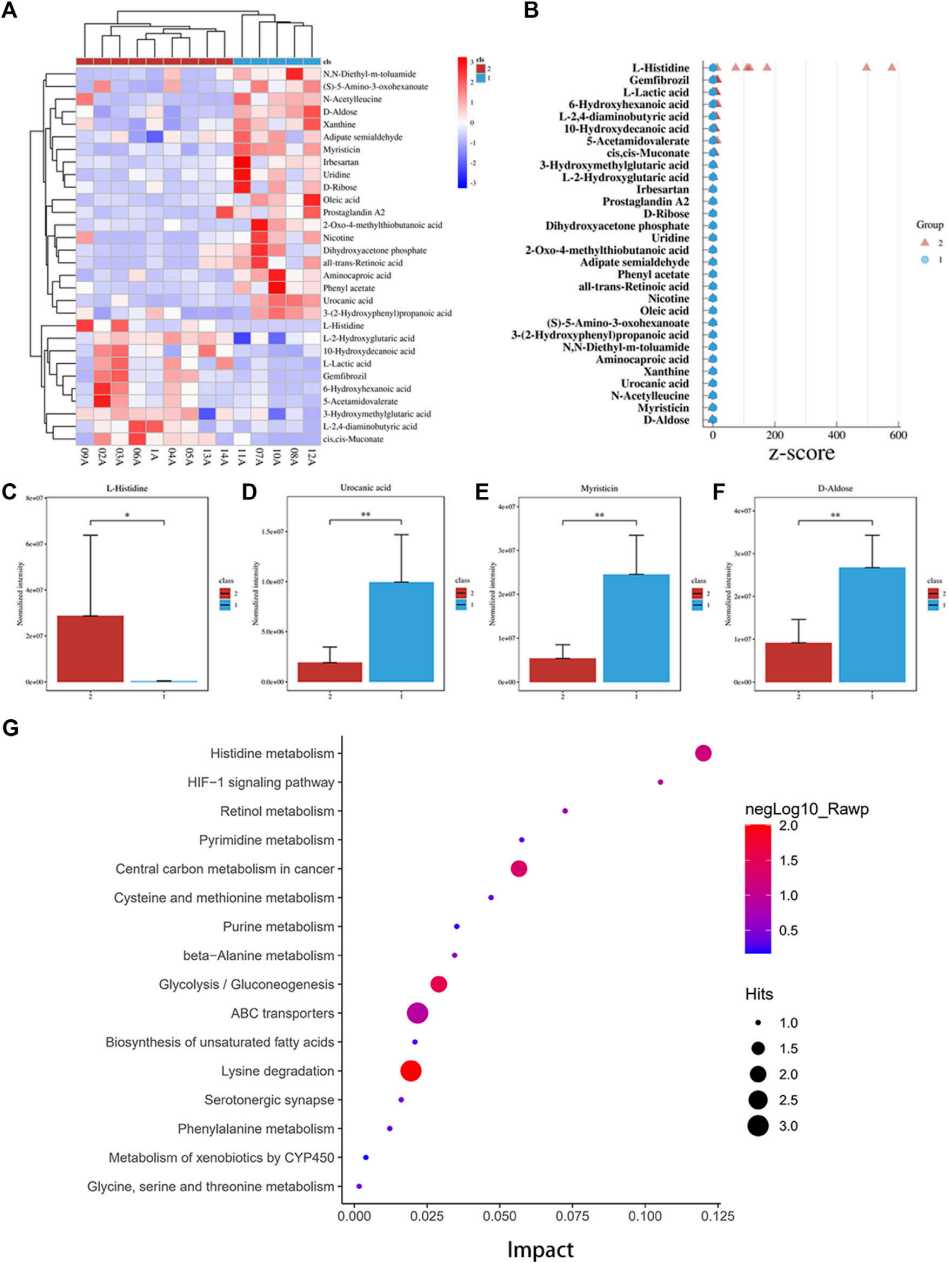
Histidine levels were significantly higher in the hypersensitivity group. **(A)** Heatmap and cluster analysis of significant differential metabolites between the normal group (group 1) and the hypersensitivity group (group 2). **(B)** Z-score graph of significant differential metabolites. l-Histidine levels in the normal group were significantly higher than those in the hypersensitivity group. **(C–F)** Four significant differential metabolites levels between the normal and hypersensitivity groups included l-histidine, urocanic acid, myristicin, and d-aldose. **(G)** Pathway enrichment analysis. Histidine metabolism showed a high impact on hypersensitivity (*p < 0.05, **p < 0.01).

To explore predictive biomarkers of PLD-induced immediate hypersensitivity, the plasma untargeted metabolomics profiles of 14 breast cancer patients before PLD were analyzed. Several metabolites showed an association with hypersensitivity, and cluster analysis indicated that the 9 allergy patients had different metabolomics profiles compared with the 5 non-allergy patients (Figure 1A; Table 2). For example, l-histidine (VIP = 1.56, fold change = 91.5, *p* value = 0.011, Figure 1C) and l-lactic acid (VIP = 1.86, fold change = 4.46, *p* value = 0.023) showed significantly higher levels in the 9 allergy patients. Additionally, myristicin (VIP = 2.96, fold change = 0.22, *p* value = 0.003), d-aldose (VIP = 2.75, fold change = 0.34, *p* value = 0.003), and urocanic acid (VIP = 2.77, fold change = 0.19, *p* value = 0.008) showed lower levels in the allergy patients (Table 2; Figures 1D–F). Z-score analysis found that l-histidine levels in particular were remarkably 91.5-fold higher in the allergy group than in the normal group (Figure 1B).

**TABLE 2:** Differential metabolites of PLD-induced HSR.

Metabolite	VIP	log2(FC_Y/N)^a^	*p* value
l-Histidine	1.5611	6.5158	0.0113
l-Lactic acid	1.8626	2.1587	0.0234
6-Hydroxyhexanoic acid	1.4778	2.1418	0.0455
10-Hydroxydecanoic acid	1.8508	1.8596	0.0455
cis,cis-Muconate	1.8400	1.5481	0.0234
5-Acetamidovalerate	1.3888	1.3339	0.0455
L-2,4-Diaminobutyric acid	1.6526	1.1169	0.0455
Gemfibrozil	1.7683	0.9827	0.0329
L-2-Hydroxyglutaric acid	2.2990	0.5882	0.0113
3-Hydroxymethylglutaric acid	1.5973	0.1013	0.0234
Adipate semialdehyde	1.7540	−0.6398	0.0455
Xanthine	2.3497	−0.8000	0.0234
Prostaglandin A2	1.3557	−1.1695	0.0455
(S)-5-Amino-3-oxohexanoate	1.6905	−1.2336	0.0329
3-(2-Hydroxyphenyl)propanoic acid	2.0955	−1.2778	0.0329
N-Acetylleucine	2.1753	−1.3983	0.0164
d-Aldose	2.7533	−1.5427	0.0034
Aminocaproic acid	2.4790	−1.6494	0.0077
Irbesartan	1.9445	−1.6648	0.0455
N,N-Diethyl-m-toluamide	2.3136	−1.8569	0.0113
Uridine	2.0579	−1.9108	0.0234
2-Oxo-4-methylthiobutanoic acid	2.1741	−1.9403	0.0455
Oleic acid	2.3902	−1.9611	0.0113
Nicotine	2.0350	−2.0880	0.0291
Myristicin	2.9585	−2.1928	0.0034
All-trans retinoic acid	2.1118	−2.2197	0.0291
Dihydroxyacetone phosphate	2.0320	−2.3603	0.0310
Urocanic acid	2.7688	−2.3720	0.0077
Phenyl acetate	2.2380	−2.6967	0.0051
d-Ribose	2.0505	−2.6990	0.0329

a FC_Y/N: fold change, yes/no.

PLD, PEGylated liposomal doxorubicin; HSR, hypersensitivity reaction.

### 3.3 MetPA Pathway Analysis

Pathway analysis was performed based on the KEGG and MetPA databases. As shown in Figure 1G, the intestinal immune network for IgA production (*p* = 0.018, compounds: all-trans retinoic acid) and the histidine metabolism pathway (*p* = 0.066, compounds: l-histidine, urocanic acid) were significantly associated with hypersensitivity. These results indicated that histidine metabolism might participate in immediate hypersensitivity.

### 3.4 Receiver Operating Characteristic Curve of Differential Metabolites

**FIGURE 2:**
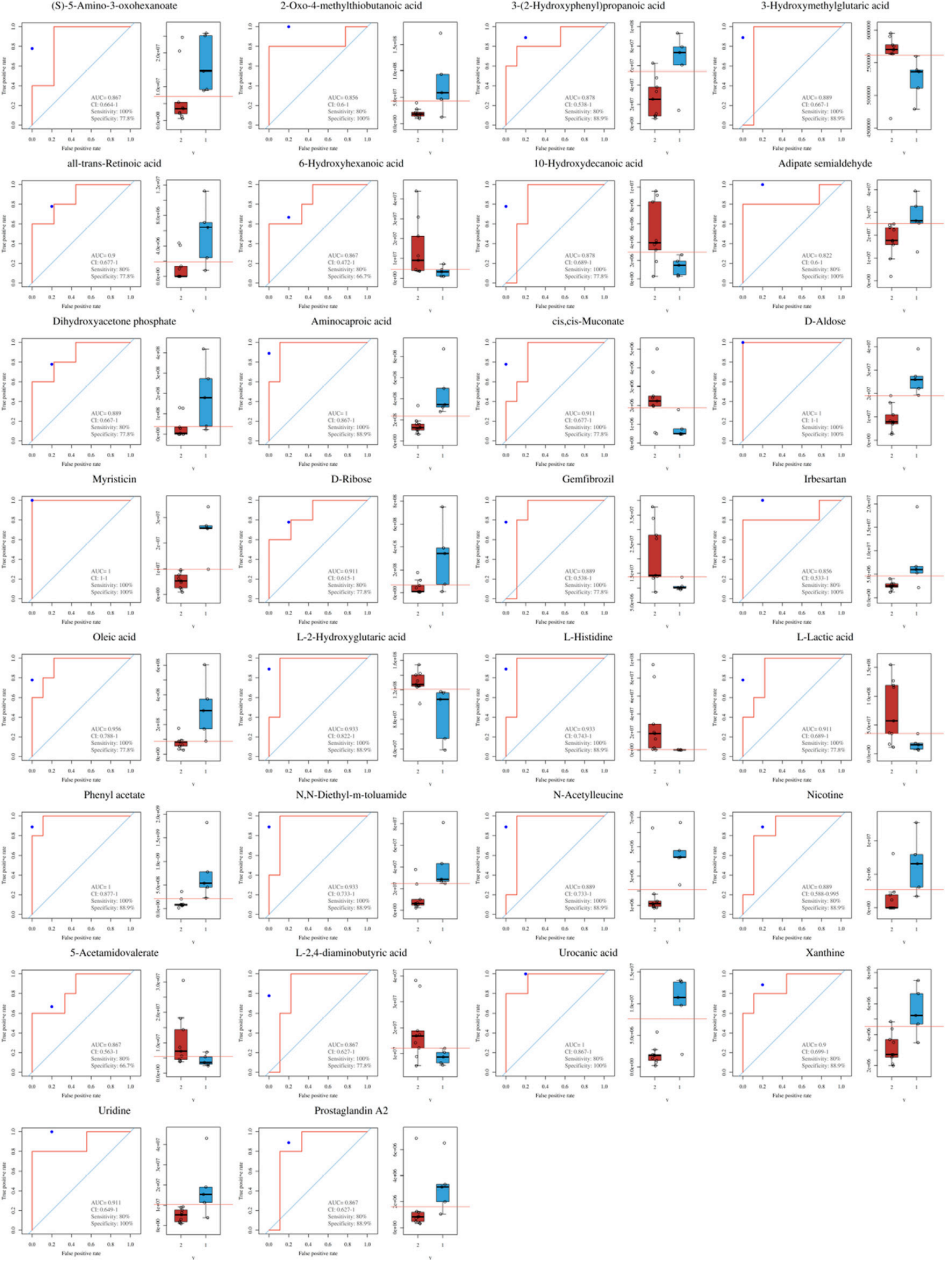
Receiver operating characteristic (ROC) curves and content level of 30 differential metabolites.

Receiver operating characteristic (ROC) analysis of differential metabolites was then calculated (Figure 2, Supplementary Table S1). The results showed that the area under the curve (AUC) of myristicin, d-aldose (AUC = 1.000, CI: 1.000–1.000, *p =* 0.023), urocanic acid (AUC = 1.00, CI: 0.867–1.000, *p =* 0.008), aminocaproic acid (AUC = 1.000, CI: 0.867–1.000, *p =* 0.008), phenyl acetate (AUC = 1.000, CI: 0.877–1.000, *p =* 0.005), oleic acid (AUC = 0.956, CI: 0.788–1.000, *p =* 0.011), l-histidine (AUC = 0.933, CI: 0.743–1.000, *p =* 0.011), L-2-hydroxyglutaric acid (AUC = 0.933, CI: 0.822–1.000, *p =* 0.011), N,N-diethyl-m-toluamide (AUC = 0.933, CI: 0.733–1.000, *p =* 0.011), uridine (AUC = 0.911, CI: 0.649–1.000, *p =* 0.023), l-lactic acid (AUC = 0.911, CI: 0.689–1.000, *p =* 0.023), cis,cis-muconate (AUC = 0.911, CI: 0.677–1.000, *p =* 0.023), and d-ribose (AUC = 0.911, CI: 0.615–1.000, *p =* 0.033) were each greater than 0.9.

### 3.5 Histidine Can Aggravate Immediate Hypersensitivity *In Vivo*

**FIGURE 3:**
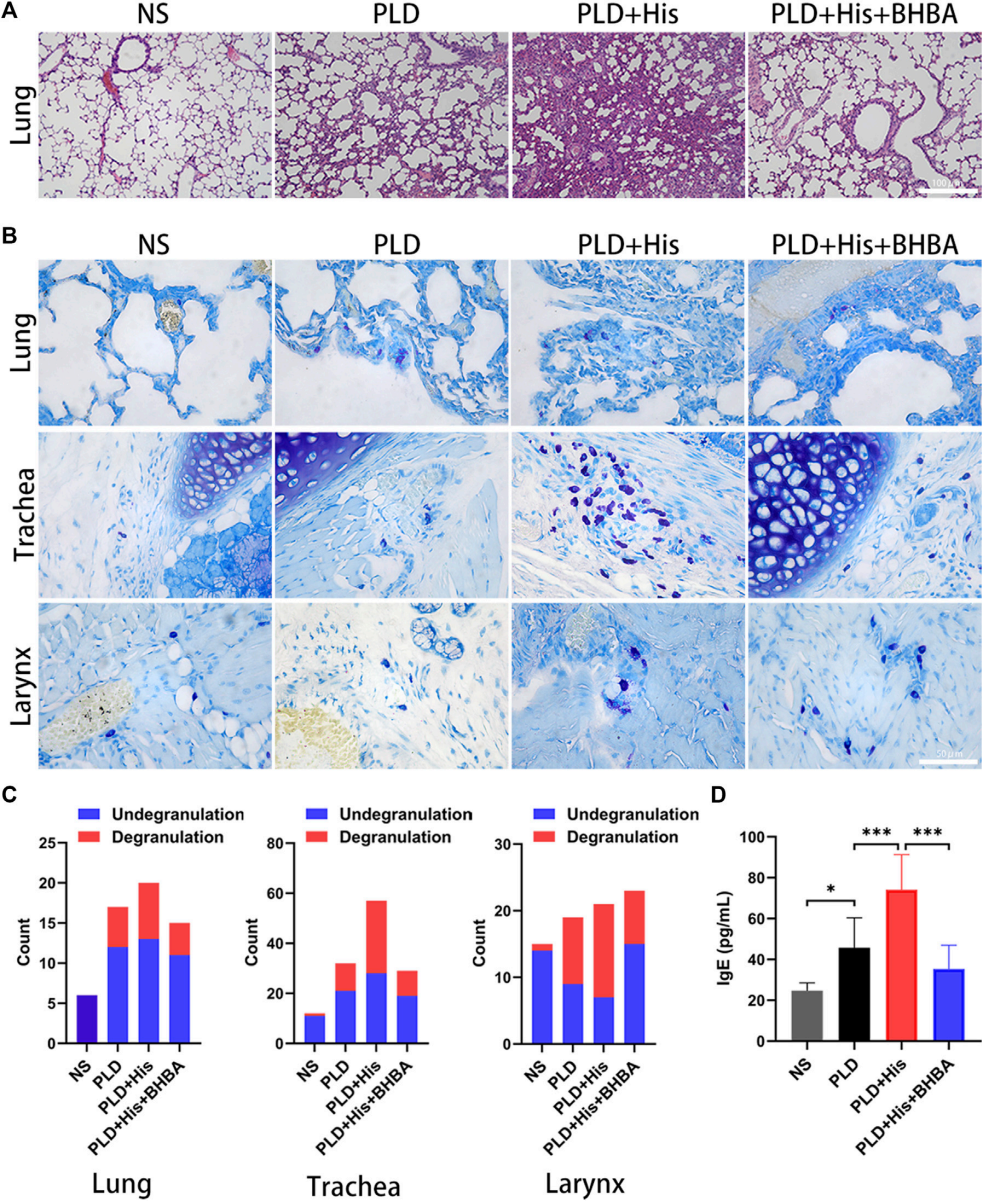
Histidine supplement-enhanced PLD-induced HSR in rat model. **(A)** H&E staining (100x). PLD injection could induce pulmonary edema. Histidine supplement-aggravated pulmonary edema, but this phenomenon could be alleviated by histidine decarboxylase inhibitor: bromo-3-hydroxybenzoic acid (BHBA). **(B)** Toluidine blue staining (mast cell staining) (200x). Histidine could increase mastocyte infiltration in the trachea and lungs and significantly increase mastocyte degranulation in the trachea and larynx after PLD injection. **(C)** Degranulation and undegranulation mast cell counting. **(D)** Histidine supplement group showed significantly increased IgE levels. BHBA treatment could decrease IgE levels significantly (PLD, PEGylated liposomal doxorubicin; HSR, hypersensitivity reaction; *p < 0.05, **p < 0.01, ***p < 0.001).

To confirm the histidine effects for immediate hypersensitivity, *in vivo* experiments were performed using a rat model. The rats showed polypnea, convulsions, and scratching of the perioral area following injection of doxorubicin liposome. The doxorubicin liposome injection could induce pulmonary edema. Furthermore, histidine supplement was found to aggravate pulmonary edema after the doxorubicin liposome injection (Figure 3A). Also, the histidine could increase the mastocyte infiltration level in the trachea and lung, as well as significantly increase mastocyte degranulation in the trachea and larynx after doxorubicin liposome injection (Figures 3B,C). As shown in Figure 3D, the histidine supplement group showed significantly increased IgE levels. However, histidine decarboxylase inhibitor 4-bromo-3-hydroxybenzoic acid (BHBA) treatment could improve the pulmonary edema, mastocyte infiltration and degranulation, and IgE levels by a significant amount. These results indicated that histidine supplement could aggravate doxorubicin liposome-induced hypersensitivity. BHBA, a histidine decarboxylase inhibitor, appeared to be effective in improving doxorubicin liposome-induced hypersensitivity symptoms and pathological damage.

## 4 Discussion

A hypersensitivity reaction (HSR) is a major adverse effect that sometimes appears after PLD injection. In this study, breast cancer patients who experienced immediate HSRs showed significantly higher l-histidine plasma levels before PLD injection. This phenomenon was later confirmed with an animal model *in vivo*. To our knowledge, this is the first metabolomics study reporting that l-histidine could be a predictive biomarker of PLD-induced immediate hypersensitivity reaction.

Premedication is usually not necessary before the administration of PLD to prevent HSRs (Yamaguchi et al., 2021). However, the incidence rate of PLD-induced HSR varies widely across existing reports. In the current study, patients experienced a fairly high incidence rate of HSRs (64.3%). In contrast, research by Alberts et al. and Yamaguchi et al. (2021) reported very low frequencies of HSRs between 7 and 9% (Alberts and Garcia, 1997). In other research, Gabizon et al. (1994) reported a frequency of 25%, while Castells et al. (2008) reported that 4 of 5 patients experienced HSR. A high percentage of HSR in this study may be caused by the small sample size. The mechanism of HSR induced by PLD is not yet known, so we first investigated and excluded systemic causes, including the injection syringe, velocity of injection flow, temperature, and environment. Chanan-Khan reported that complement activation may play a key role in HSRs induced by PLD. Polyethylene glycol (PEG) is one of the pharmaceutical adjuvants of PLD. Chen et al. (2020) showed that PLD vesicle damage and doxorubicin release were triggered by anti-PEG antibody and mediated by the complement terminal complex (Gabizon and Szebeni, 2020). This might be a potential mechanism of HSR but was not evaluated by the clinical study. Also, immediate hypersensitivity is induced by IgE. But in the current study, no significant association was found between plasma IgE, complement 3 (C3), and complement 4 (C4) levels and HSRs (Table 1). Therefore, to explore potential biomarkers and mechanisms of PLD-induced HSRs, a comprehensive untargeted metabolomics analysis of plasma obtained before PLD injection was performed. l-Histidine showed the most significantly higher level in HSR patients (VIP = 1.56, fold change = 91.5, *p* value = 0.011, Figure 1C).

Histidine is an amino acid that plays an important role in the scavenging of reactive oxygen and nitrogen species, erythropoiesis, and the histaminergic system (Holeček, 2020). Histamine, a “shock toxin” (SCHAYER, 1960), is metabolized from l-histidine by histidine decarboxylase (HDC) (SCHAYER, 1962; Holeček, 2020). Histamine is involved in shock (SCHAYER, 1960; Yamani et al., 2018), allergies (Church, 2017; Yang and Kim, 2019), and atopic dermatitis (Palmer et al., 2006). Most histamine is presynthesized and stored in granules in mast cells and basophils (Holeček, 2020). Histamine will release *via* degranulation after immunological stimulation (Holeček, 2020). Also, dietary histidine affects histamine concentration in immune cells and alters immune system function and anaphylactic reaction (Lee et al., 1981; Yoshimatsu et al., 2002; Holeček, 2020). Histidine can be metabolized to histamine by gastrointestinal microbiota such as all *M. morganii* strains and some *L. reuteri* strains (Chen et al., 2019). For this study, it was supposed that high histidine levels would increase histamine levels and could thus be a trigger or enhancer for HSR. In the rat model, the l-histidine supplement group showed more serious polypnea, convulsions, and scratching of the perioral area after doxorubicin liposome injections. Meanwhile, l-histidine significantly increased pulmonary edema levels (Figure 3A), mastocyte infiltration, and degranulation in the trachea, larynx, or lung tissue after doxorubicin liposome injection (Figures 3B,C). Furthermore, these phenomena could be rescued by BHBA, an HDC inhibitor. These results revealed that a high l-histidine level was a risk biomarker and HSR enhancement factor in patients who received PLD. High l-histidine levels might increase histamine storage in mast cells and lead to severe HSR. The mechanism of HSR induced by PLD will be further clarified in an upcoming, follow-up study.

Antihistamine therapy or HDC inhibitor and limiting dietary histidine could be effective methods to prevent PLD-induced HSRs. Diphenhydramine, or any other antihistamine drug, is used to treat HSRs or other allergy reactions. The HDC inhibitor BHBA could be a potential prophylactic treatment for PLD or other liposomal drug. Adults can consume histidine from food such as chicken breast (Sato et al., 2008). As such, limiting dietary histidine before PLD injection may need to be considered.

There are several limitations to this study. First, the sample size of this research was not sufficient to verify the results. Considering sample size limitation, we designed an *in vivo* experiment in rats to confirm the effects of histidine. A larger sample size and prospective trial should be performed in the future. Second, while this research found and proved that high l-histidine levels would enhance HSR *in vivo*, it did not investigate the underlying mechanism. The fact that l-histidine enhances HSR by increasing histamine storage is not yet proven. Uncovering this mechanism will help improve PLD clinical side effect management. Third, untargeted metabolomics analysis is a relative quantification method. A sensitive, specific LC-MS/MS method should be established to detect histidine in the future.

This study is the first known report that l-histidine could be a risk biomarker and enhancer for PLD-induced HSR. Our research suggests that antihistamine therapy and l-histidine dietary management should be emphasized in PLD-treated patients. Moreover, these findings support metabolomics study may serve as a new strategy for the treatment and mechanism exploration of side effects.

## Data Availability

The original contributions presented in the study are included in the article/Supplementary Materials, further inquiries can be directed to the corresponding authors.
