# Supplementary material for: Biomarkers of PEGylated Liposomal Doxorubicin-Induced Hypersensitivity Reaction in Breast Cancer Patients Based on Metabolomics
Source: Front Pharmacol. 2022 Apr 21;13:827446. doi: 10.3389/fphar.2022.827446 (PMC9068896; doi:10.3389/fphar.2022.827446)
Supplement: Supplementary file 1 [file DataSheet2.docx]

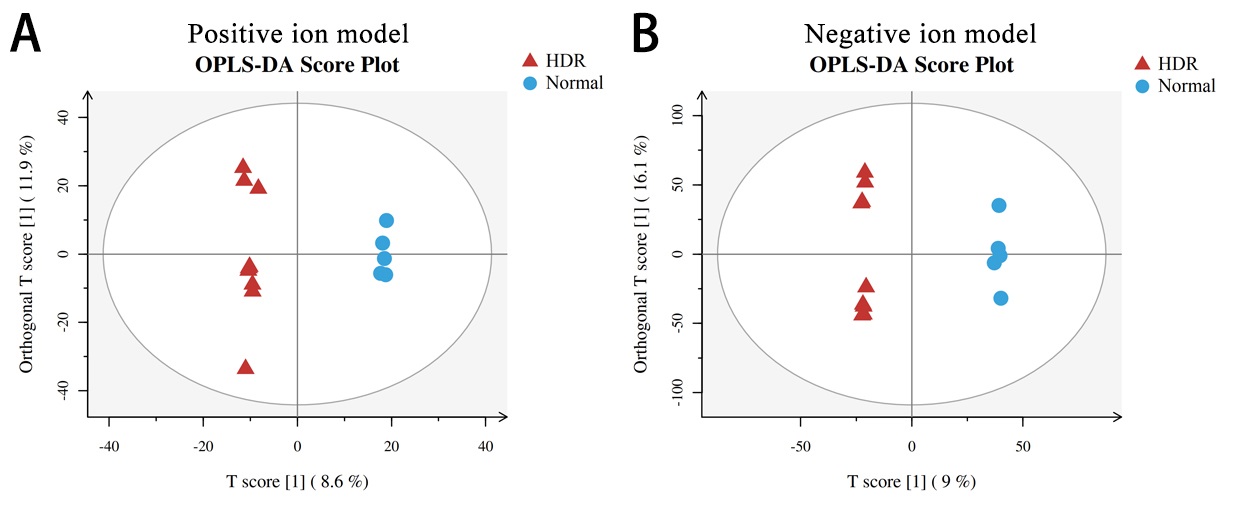


**Supplement Figure 1. OPLS-DA models for separating HSR and non-HSR patients. (A)** OPLS-DA plot for positive ion model. The respective model interpretability for the X and Y variable datasets was R2X=0.346 cum, R2Y=0.996 cum, and model predictability Q2=0.323 cum. **(B)** OPLS-DA plot for negative ion model. R2X=0.421 cum, R2Y=0.999 cum, and model predictability Q2=0.344 cum.


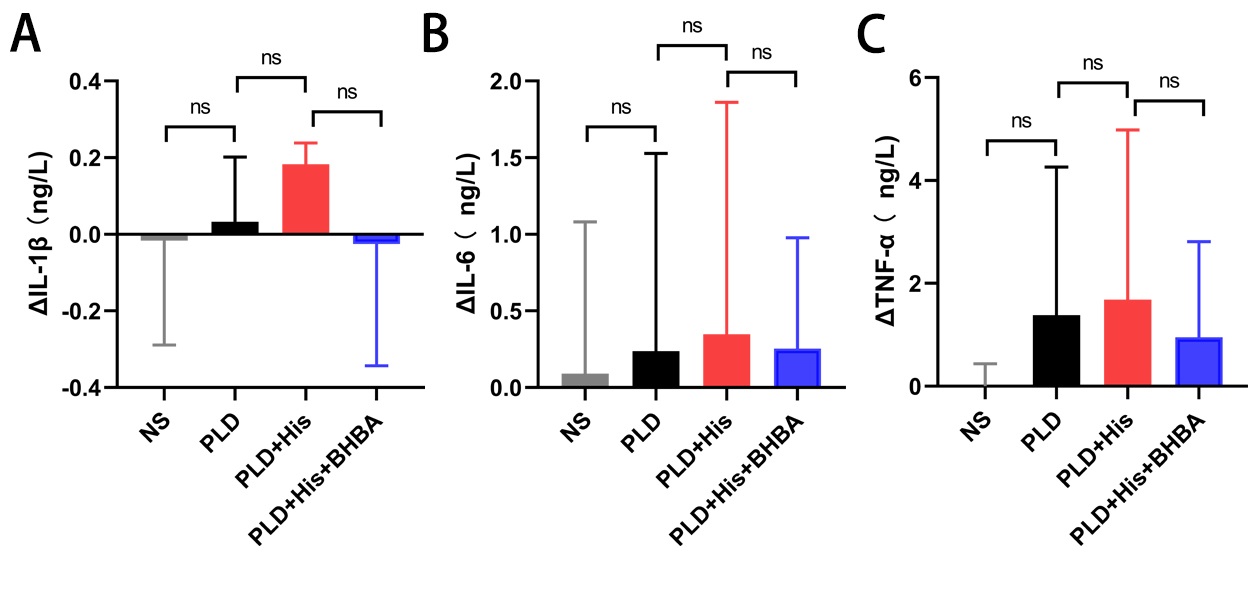


**Suppelement Figure 2. Cytokines change 2 minutes after PLD injection in Rats.** A.ΔIL-1β; B. ΔIL-6; C.ΔTNF-α. Mean±SD (n=3).
