# Supplementary material for: Biomarkers of PEGylated Liposomal Doxorubicin-Induced Hypersensitivity Reaction in Breast Cancer Patients Based on Metabolomics
Source: Front Pharmacol. 2022 Apr 21;13:827446. doi: 10.3389/fphar.2022.827446 (PMC9068896; doi:10.3389/fphar.2022.827446)
Supplement: Supplementary file 2 [file DataSheet1.docx]

**Supplement Table 1** CTCAE classification of doxorubicin liposomes induced side effects in 14 patients.

| Patient ID | Hypersensitivity | IgE* (IU/mL) | C3^#^ (mg/L) | C4† (mg/L) |
| --- | --- | --- | --- | --- |
| A01 | 2 | 167 | 1510 | 486 |
| A02 | 2 | 225 | 1090 | 188 |
| A03 | 2 | 4 | 714 | 154 |
| A04 | 2 | 18 | 1310 | 258 |
| A05 | 2 | 413 | 1060 | 365 |
| A06 | 2 | 85 | 1100 | 236 |
| A07 | 2 | 81 | 1180 | 281 |
| A08 | 0 | 7 | 1250 | 239 |
| A09 | 0 | 20 | 1210 | 275 |
| A10 | 2 | 4 | 1170 | 532 |
| A11 | 0 | 42 | 1340 | 289 |
| A12 | 0 | 12 | 1020 | 321 |
| A13 | 0 | 12 | 1170 | 399 |
| A14 | 2 | 4 | 1450 | 431 |

CTCAE: Common Terminology Criteria for Adverse Events; * IgE normal range (0-100 IU/mL); # C3 normal range (650-1800 mg/L); † C4 normal range (100-400 mg/L).

**Supplement Table 2** ROC details of difference metabolite in 14 patients

| Metabolite | AUC | Specificity | Sensitivity | Threshold |
| --- | --- | --- | --- | --- |
| Myristicin | 1.000 | 1.000 | 1.000 | 9761961.2 |
| D-Aldose | 1.000 | 1.000 | 1.000 | 19132289.1 |
| Urocanic acid | 1.000 | 1.000 | 0.800 | 7624731.8 |
| Aminocaproic acid | 1.000 | 0.889 | 1.000 | 235238952.6 |
| Phenyl acetate | 1.000 | 0.889 | 1.000 | 214703645.4 |
| Oleic acid | 0.956 | 0.778 | 1.000 | 87556259.3 |
| L-Histidine | 0.933 | 0.889 | 1.000 | 646211.2 |
| L-2-Hydroxyglutaric acid | 0.933 | 0.889 | 1.000 | 120912442.0 |
| N,N-Diethyl-m-toluamide | 0.933 | 0.889 | 1.000 | 24525731.0 |
| Uridine | 0.911 | 1.000 | 0.800 | 10335362.3 |
| L-Lactic acid | 0.911 | 0.778 | 1.000 | 35136670.2 |
| cis,cis-Muconate | 0.911 | 0.778 | 1.000 | 1869045.1 |
| D-Ribose | 0.911 | 0.778 | 0.800 | 69421543.0 |
| Xanthine | 0.900 | 0.889 | 0.800 | 4532447.5 |
| all-trans-Retinoic acid | 0.900 | 0.778 | 0.800 | 1895077.6 |
| N-Acetylleucine | 0.889 | 0.889 | 1.000 | 2094942.4 |
| 3-Hydroxymethylglutaric acid | 0.889 | 0.889 | 1.000 | 5615714.3 |
| Nicotine | 0.889 | 0.889 | 0.800 | 2718322.5 |
| Gemfibrozil | 0.889 | 0.778 | 1.000 | 13807408.4 |
| Dihydroxyacetone phosphate | 0.889 | 0.778 | 0.800 | 37672542.9 |
| 3-(2-Hydroxyphenyl)propanoic acid | 0.878 | 0.889 | 0.800 | 53949535.8 |
| 10-Hydroxydecanoic acid | 0.878 | 0.778 | 1.000 | 2894222.1 |
| Prostaglandin A2 | 0.867 | 0.889 | 0.800 | 1625187.5 |
| L-2,4-diaminobutyric acid | 0.867 | 0.778 | 1.000 | 12107330.1 |
| (S)-5-Amino-3-oxohexanoate | 0.867 | 0.778 | 1.000 | 7117591.8 |
| 5-Acetamidovalerate | 0.867 | 0.667 | 0.800 | 5519070.1 |
| 6-Hydroxyhexanoic acid | 0.867 | 0.667 | 0.800 | 4337622.9 |
| 2-Oxo-4-methylthiobutanoic acid | 0.856 | 1.000 | 0.800 | 43363251.7 |
| Irbesartan | 0.856 | 1.000 | 0.800 | 4646098.6 |
| Epiandrosterone | 0.844 | 0.667 | 0.800 | 176858164.0 |
| Adipate semialdehyde | 0.822 | 1.000 | 0.800 | 25124054.2 |
| N-Acetyl-L-phenylalanine | 0.822 | 0.667 | 0.800 | 2189217.2 |
| Kynurenic acid | 0.822 | 0.556 | 1.000 | 228371.8 |
| 1,2,3-Trihydroxybenzene | 0.778 | 0.889 | 0.600 | 11103214.9 |
| Dibutyl phthalate | 0.778 | 0.778 | 0.800 | 4542872.8 |
| 5,6-DHET | 0.744 | 0.778 | 0.600 | 1913775.3 |
| Hydrogen phosphate | 0.744 | 0.667 | 1.000 | 416695004.0 |
| Alpha-D-Glucose | 0.733 | 0.889 | 0.600 | 2929104.0 |
| Norepinephrine | 0.733 | 0.667 | 1.000 | 68735282.4 |
| Phenylacetaldehyde | 0.733 | 0.667 | 1.000 | 947495.6 |
| beta-Alanine | 0.722 | 0.778 | 0.600 | 66382816.0 |
| Mandelic acid | 0.700 | 0.667 | 0.800 | 8003393.9 |
| 3-Methyladenine | 0.689 | 0.667 | 0.800 | 50647718.0 |
| p-Hydroxyphenylacetic acid | 0.678 | 0.667 | 0.800 | 95214964.1 |
| Cytosine | 0.667 | 0.667 | 0.800 | 1648805.5 |
| Biochanin A | 0.667 | 0.556 | 1.000 | 2132808.2 |
| 4-Quinolinecarboxylic acid | 0.633 | 0.556 | 0.800 | 1768236.7 |
| Maleic acid | 0.611 | 0.444 | 0.800 | 4985011.0 |
| Caffeate | 0.600 | 0.556 | 0.800 | 27848364.0 |
| 3,3-Dimethoxybenzidine | 0.600 | 0.556 | 0.800 | 955972.4 |
| Capric acid | 0.578 | 0.556 | 1.000 | 14787563.8 |
| 3-Amino-4-hydroxybenzoate | 0.533 | 0.667 | 0.600 | 15155894.5 |
| Benzamide | 0.522 | 0.444 | 0.800 | 14884.0 |
| Maltol | 0.444 | 0.556 | 0.800 | 15087467.5 |
| Cimetidine | 0.400 | 0.778 | 0.600 | 87269.6 |

Supplement Table 3. PLD injection and drug combination details of 14 patients.

| **Patient ID** | **Breakfast time and menu** | **Sample collection time** | **PLD injection time** | **Drug combination before PLD administration** | **HDR** |
| --- | --- | --- | --- | --- | --- |
| 1 | 2021.02.24, 07:00,  Pork noodles | 2021.02.24 09:52 | 2021.02.24  10:00 | NA | Yes |
| 2 | 2021.03.26, 07:43,  Pork noodles | 2021.03.26 09:41 | 2021.03.26,  10:00 | NA | Yes |
| 3 | 2021.02.25, 07:10,  Pork noodles | 2021.02.25 09:25 | 2021.02.25,  09:35 | NA | Yes |
| 4 | 2021.03.05, 07:02,  Pork noodles | 2021.03.05 09:19 | 2021.03.05,  09:45 | NA | Yes |
| 5 | 2021.03.19, 07:05,  Pork noodles | 2021.03.19 09:24 | 2021.03.19,  09:35 | NA | Yes |
| 6 | 2021.01.21, 07:03,  Pork noodles | 2021.01.21 09:43 | 2021.01.21,  09:55 | 2021.01.20 Tramadol, 100mg, once, p.o. | No |
| 7 | 2021.01.27, 07:11,  Pork noodles | 2021.01.27 09:40 | 2021.01.27,  10:00 | NA | Yes |
| 8 | 2021.02.05, 07:00,  Pork noodles | 2021.02.05 09:23 | 2021.02.05,  09:40 | NA | No |
| 9 | 2021.02.07, 08:27,  Pork noodles | 2021.02.07 10:29 | 2021.02.07,  10:40 | 2021.02.06, Rivaroxaban, 10mg, Q.d., p.o. | Yes |
| 10 | 2020.12.17, 07:08,  Pork noodles | 2020.12.17 09:20 | 2020.12.17,  09:31 | NA | No |
| 11 | 2020.12.23, 06:37,  Pork noodles | 2020.12.23 09:22 | 2020.12.23,  09:26 | 2020.12.15, Recombinant Human Granulocyte Colony-stimulating Factor, 150 μg, Q.d., i.h. | No |
| 12 | 2021.01.05, 06:40, Pork noodles | 2021.01.05 09:26 | 2021.01.05,  09:31 | Long term. Irbesartan hydrochlorothiazide, 150mg, Q.d., p.o. | No |
| 13 | 2021.01.12, 07:12,  Pork noodles | 2021.01.12 09:48 | 2021.01.12,  09:55 | 2021.01.05, Metoprolol Succinate, 47.5mg, Q.d., p.o.,  and Irbesartan hydrochlorothiazide, 50mg, Q.d., p.o. | Yes |
| 14 | 2021.01.12, 07:11,  Pork noodles | 2021.01.12 09:54 | 2021.01.12,  10:10 | NA | Yes |
